# Supplementary material for: Temporal and genetic variation in female aggression after mating
Source: PLoS One. 2020 Apr 29;15(4):e0229633. doi: 10.1371/journal.pone.0229633 (PMC7190144; doi:10.1371/journal.pone.0229633)
Supplement: S1 Fig — Colours indicate the genotype of the male that a female mated with–blue = Canton-S, yellow = Dahomey, red = w1118, empty circle = virgin female. Black bars indicate treatment means ± 1 standard error. (DOCX) [file pone.0229633.s001.docx]

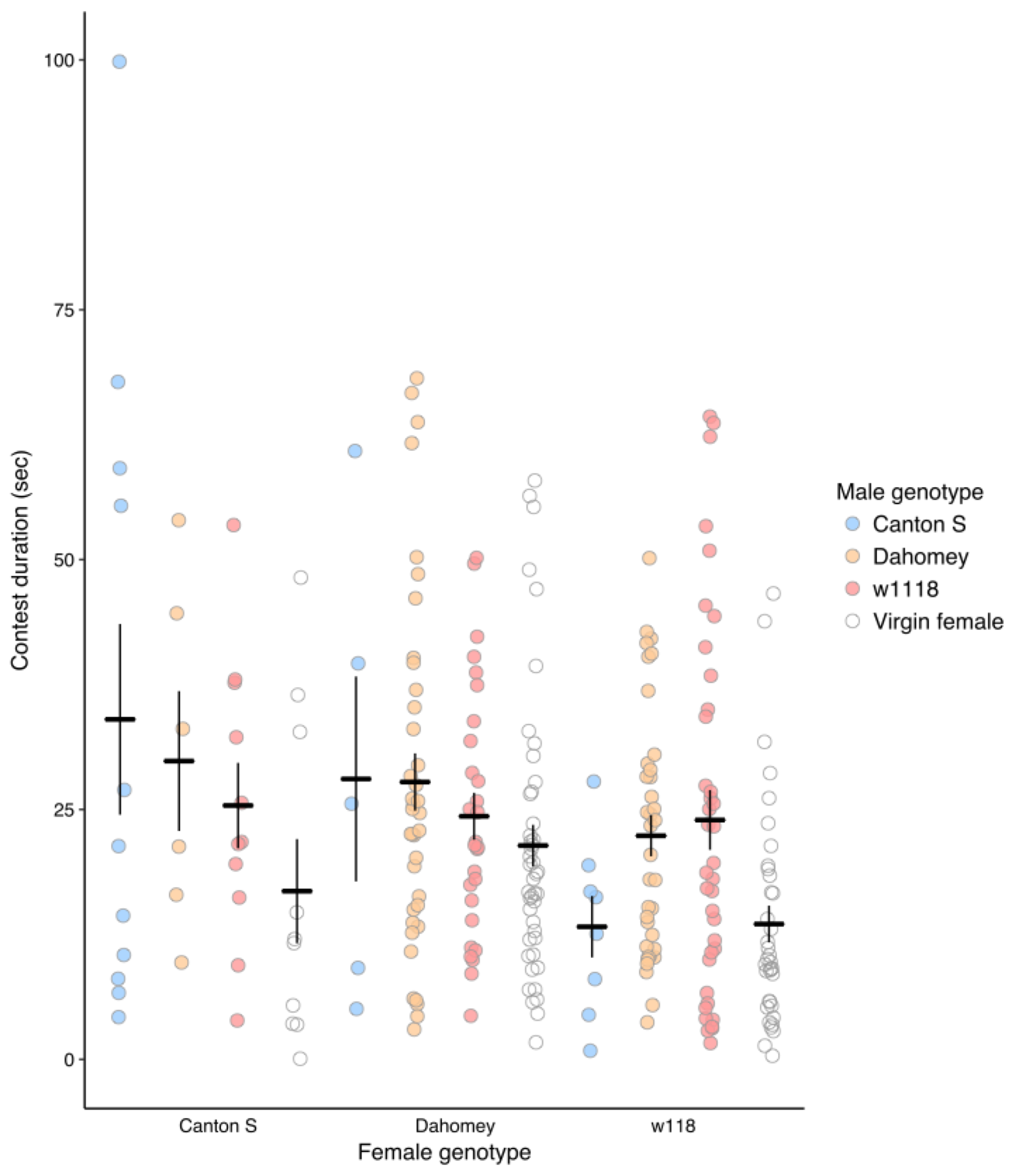


**Supplementary Figure 1: Mated females fight for longer than virgin females but this does not depend on the genotype of their mate**

Colours indicate the genotype of the male that a female mated with – blue = Canton-S, yellow = Dahomey, red = *w^1118^*, empty circle = virgin female. Black bars indicate treatment means ± 1 standard error.
