# Supplementary material for: Temporal and genetic variation in female aggression after mating
Source: PLoS One. 2020 Apr 29;15(4):e0229633. doi: 10.1371/journal.pone.0229633 (PMC7190144; doi:10.1371/journal.pone.0229633)
Supplement: S4 Fig — Colours indicate the genotype of the female–blue = Canton-S, yellow = Dahomey, red = w1118. Black bars indicate treatment means ± 1 standard error. All females used in contests were included in this figure. (DOCX) [file pone.0229633.s004.docx]

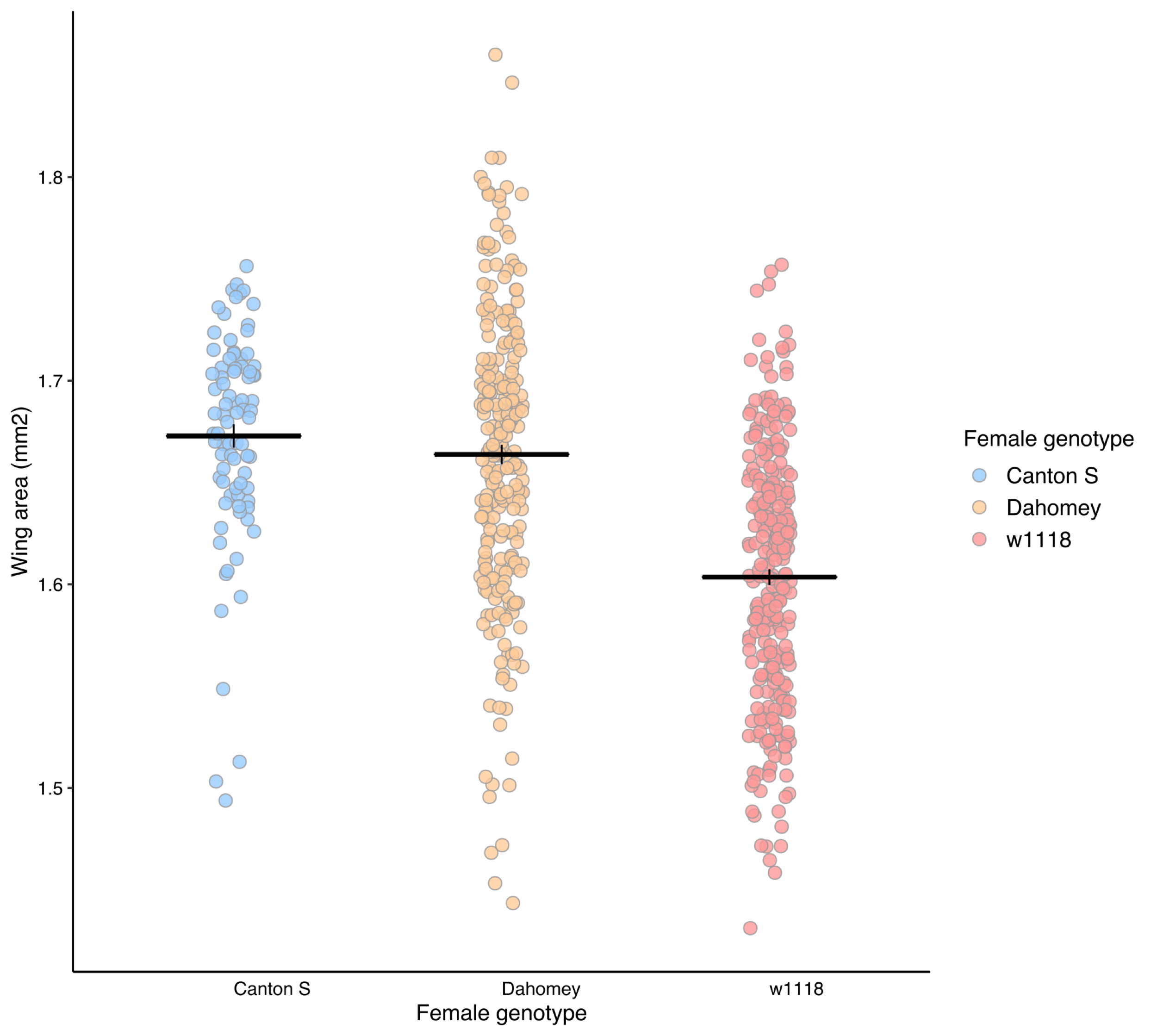


**Supplementary Figure 4:** *w^1118^* **females had smaller wings than Dahomey and Canton S**

Colours indicate the genotype of the female – blue = Canton-S, yellow = Dahomey, red = *w^1118^*. Black bars indicate treatment means ± 1 standard error. All females used in contests were included in this figure.
