# Supplementary material for: Temporal and genetic variation in female aggression after mating
Source: PLoS One. 2020 Apr 29;15(4):e0229633. doi: 10.1371/journal.pone.0229633 (PMC7190144; doi:10.1371/journal.pone.0229633)
Supplement: S7 Fig — This figure shows the setup we used for contests between females, which consisted of a circular arena with a food cap set into the middle. This food cap contained regular fly food medium, with a drop of yeast paste in the middle to act as a valuable, restricted resource. In this image, you can see a shadow that covers the top portion of the arena, which influences the reliability of the tracking software. You can also see the restricted contrast between flies on the food cap compared with flies on the white background, which again makes it difficult for the tracking software to detect the flies accurately. The label at the top of the screen indicates the treatment, which was covered during observations and manual scoring of behaviors. (DOCX) [file pone.0229633.s007.docx]

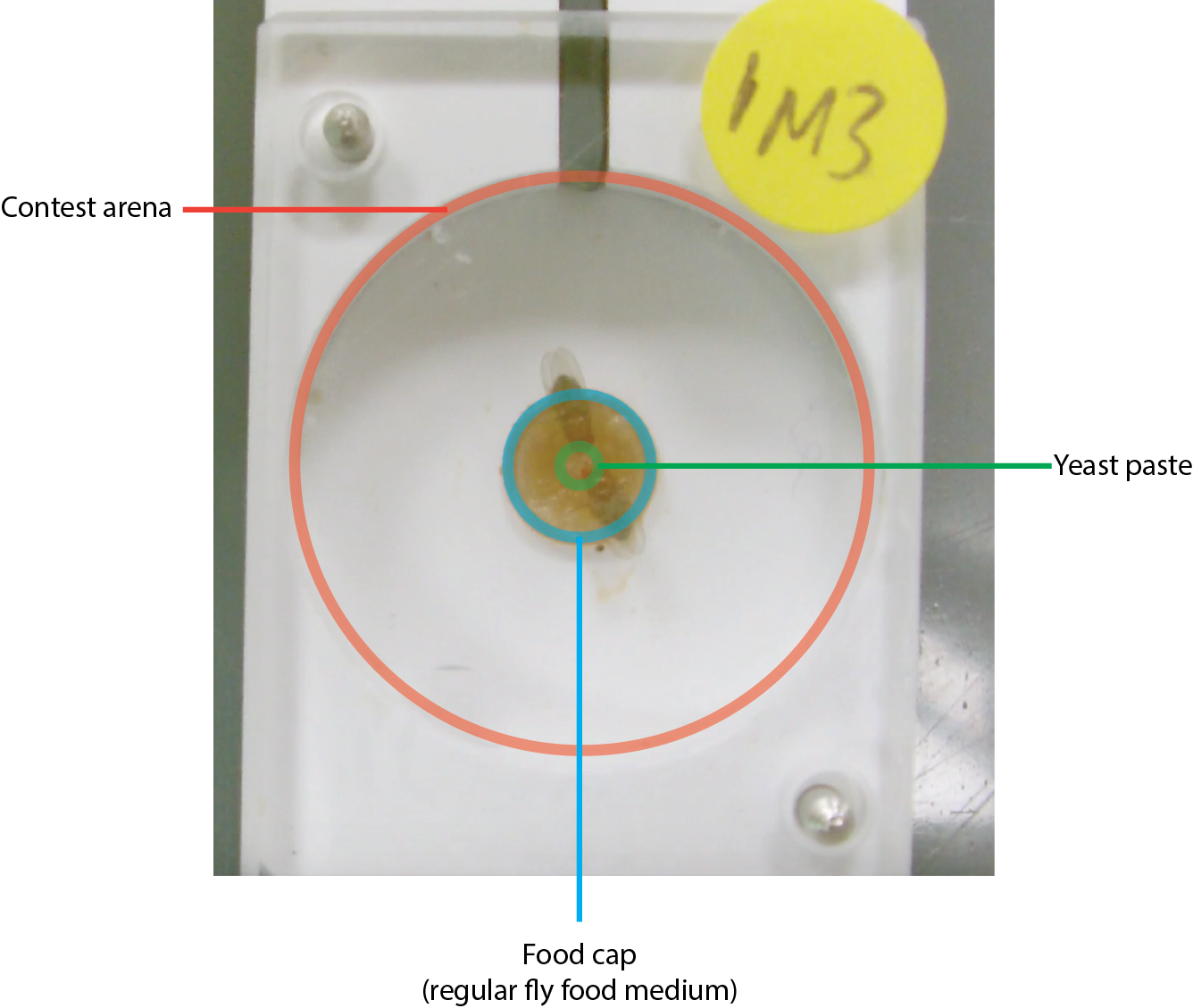


**Supplementary Figure 7: Contest arena setup**

This figure shows the setup we used for contests between females, which consisted of a circular arena with a food cap set into the middle. This food cap contained regular fly food medium, with a drop of yeast paste in the middle to act as a valuable, restricted resource. In this image, you can see a shadow that covers the top portion of the arena, which influences the reliability of the tracking software. You can also see the restricted contrast between flies on the food cap compared with flies on the white background, which again makes it difficult for the tracking software to detect the flies accurately. The label at the top of the screen indicates the treatment, which was covered during observations and manual scoring of behaviors.
