# Supplementary material for: Temporal and genetic variation in female aggression after mating
Source: PLoS One. 2020 Apr 29;15(4):e0229633. doi: 10.1371/journal.pone.0229633 (PMC7190144; doi:10.1371/journal.pone.0229633)
Supplement: S1 Table — Sample sizes indicate the number of pairs that the tracking software successfully tracked for each treatment in the genotypes experiment. (DOCX) [file pone.0229633.s008.docx]

**Supplementary Table 1: Sample sizes for genotype experiment**

Sample sizes indicate the number of pairs that the tracking software successfully tracked for each treatment in the genotypes experiment.

|  | **Female genotype** | | | |
| --- | --- | --- | --- | --- |
| **Male genotype** | **Canton-S** | **Dahomey** | ***w^1118^*** | **Total** |
| Canton-S | 11 | 5 | 8 | 24 |
| Dahomey | 6 | 38 | 36 | 80 |
| *w^1118^* | 11 | 28 | 38 | 77 |
| Virgins | 10 | 45 | 36 | 91 |
| **Total** | **38** | **116** | **118** | **272** |
