# Supplementary material for: Temporal and genetic variation in female aggression after mating
Source: PLoS One. 2020 Apr 29;15(4):e0229633. doi: 10.1371/journal.pone.0229633 (PMC7190144; doi:10.1371/journal.pone.0229633)
Supplement: S2 Table — Sample sizes indicate number of pairs in each treatment. The number of pairs that were scored manually (the total number of pairs for that treatment) is recorded in the first column, while the number of pairs that were scored using automated tracking and analysis software are in the second column for both mated and virgin pairs. (DOCX) [file pone.0229633.s009.docx]

**Supplementary Table 2: Sample sizes for timing experiment**

Sample sizes indicate number of pairs in each treatment. The number of pairs that were scored manually (the total number of pairs for that treatment) is recorded in the first column, while the number of pairs that were scored using automated tracking and analysis software are in the second column for both mated and virgin pairs.

|  | **Mated** | | **Virgin** | |
| --- | --- | --- | --- | --- |
| **Hour after mating** | **Manual** | **Tracked** | **Manual** | **Tracked** |
| **1** | 17 | 16 | 16 | 14 |
| **2** | 16 | 8 | 18 | 14 |
| **4** | 23 | 20 | 22 | 16 |
| **8** | 13 | 11 | 13 | 13 |
| **24** | 21 | 20 | 22 | 19 |
| **168** | 22 | 16 | 24 | 22 |
| **Total** | **112** | **91** | **115** | **98** |
