# Supplementary material for: Temporal and genetic variation in female aggression after mating
Source: PLoS One. 2020 Apr 29;15(4):e0229633. doi: 10.1371/journal.pone.0229633 (PMC7190144; doi:10.1371/journal.pone.0229633)
Supplement: S1 File — (DOCX) [file pone.0229633.s010.docx]

**Temporal and genetic variation in female aggression after mating**

**Supplementary Information**

Additional results for the genotype experiment

*Mating duration*

Female genotypes did not differ in mating duration (LM: F_2,422_ = 0.18, P = 0.72; Fig. 3). *w^1118^* males mated for less time than Canton-S or Dahomey males (F_2,422_ = 2.62, P = 0.001). There was no significant interaction between male and female genotype (F_4,422_ = 0.78, P = 0.67).

*Mating latency*

We tested latency to mate using a Cox proportional hazards model from the R package ‘survival’ (1). We found significant effects of female genotype (log likelihood = -2179.8, P < 0.001), male genotype (log likelihood = -2091.9, P < 0.001), and an interaction between the two (log likelihood = -2081, P < 0.001). *w^1118^* males were much slower to mate than Canton S or Dahomey males, while Canton S females were quicker to mate than Dahomey or *w^1118^* females.

*Wing area*

As an indicator of body size, we measured wing area of all females fought in the genotype experiment (2). We removed one outlier in the *w^1118^* treatment (identified using a Bonferroni outlier test in the ‘car’ package in R; (3)). *w^1118^* females had significantly smaller wings than either Canton-S or Dahomey females (Supp. Fig. 4; LM: F_2,588_ = 67.11, P < 0.0001; Multiple comparisons: Dah- *w^1118^*: log diff = 0.06, P < 0.0001; CS- *w^1118^*: log diff = 0.07, P < 0.0001; CS-Dah: log diff = 0.009, P = 0.52).

*Egg counts*

We collected eggs from females in the genotype experiment that they laid in the 24 hours between mating and fighting, and in the 24 hours after fighting. As body size can influence the number of eggs females lay (2,4), we included wing area as a covariate in our analysis of egg counts. We conducted two separate analyses on each set of egg data:

1. Testing whether female genotype, mating status, and wing area determined how many eggs females laid (including both mated and virgin females).
2. Testing whether male genotype and female genotype, and wing area determined how many eggs mated females laid.

Egg production in overnight vials between mating and fighting

1. We found significant effects of female genotype (Dev_2,579_ = 728.36, P < 0.0001), mating status (Dev_1,579_ = 1642.24, P < 0.001), and wing size (Dev_1,579_ = 57.49, P = 0.005) on pre-fighting egg counts. Canton S females laid significantly fewer eggs than Dahomey and *w^1118^* females, while mated females laid more eggs than virgins (Supp. Fig. 5). There were no significant interactions (Mating status * female genotype: Dev_2,579_ = 6.32, P = 0.65; Mating status * wing area: Dev_1,579_ = 4.47, P = 0.44; Female genotype * wing area: Dev_2,579_ = 13.02, P = 0.42; Mating status * female genotype * wing area: Dev_2,579_ = 0.27, P = 0.98).

2. We again found significant effects of female genotype (Dev_2,392_ = 700.27, P < 0.001), and wing size (Dev_1,392_ = 47.02, P = 0.01) on pre-fighting egg count for mated females. There was no significant effect of male genotype (Dev_2,392_ = 10.91, P = 0.48) on egg count. Nor were there any significant interactions (Female genotype * male genotype: Dev_4,392_ = 27.76, P = 0.45; Female genotype * wing area: Dev_2,392_ = 9.85, P = 0.52; Female genotype * male genotype * wing area: Dev_4,392_ = 10.84, P = 0.84), although there was a marginally non-significant interaction between male genotype and wing area (Dev_2,396_ = 39.39, P = 0.07), whereby there was a positive relationship between female wing size and egg count, but only in females mated to Canton S males.

Overnight vials after fighting

1. In our first model, there were two significant interactions. The first was between female genotype and mating status, where there was a greater difference between mated and virgin Canton-S females than there was in Dahomey and *w^1118^* females (GLM with quasipoisson distribution: Dev_2, 580_ = 272.4, P < 0.001; Supp. Fig. 6). The second was that there was a different relationship between mating status and wing area for mated and virgin females, with wing area appearing to be more positively correlated with the number of eggs laid in mated females than in virgin females (Dev_1, 580_ = 43.30, P = 0.01). No other interactions were significant (Female genotype * wing area: Dev_2, 580_ = 4.82, P = 0.71; Female genotype * mating status * wing area: Dev_2, 580_ = 15.8, P = 0.33). In addition, there were main effects of female genotype (Dev_2, 589_ = 162.81, P < 0.001), mating status (Dev_1, 588_ = 2300.32, P < 0.001), and wing area (Dev_1, 587_ = 112.8, P < 0.001).

2. Female genotype was again significant (GLM with quasipoisson distribution: Dev_2, 392_ = 103.66, P < 0.001). Wing area was also significant, with larger females consistently laying more eggs (Dev_1, 392_ = 148.03, P < 0.001). Male genotype was not significant, nor were any of the interactions (Male genotype: Dev_2, 392_ = 16.95, P = 0.25; Female genotype * male genotype: Dev_4, 392_ = 10.32, P = 0.79; Female genotype * wing area: Dev_2, 392_ = 2.01, P = 0.85; Male genotype * wing area: Dev_2 392_ = 26.39, P = 0.11; Female genotype * male genotype * wing area: Dev_4, 392_ = 33.15, P = 0.24).

**The use of automated tracking and classification software**

We tested whether automated tracking and classification software could be used in our existing setup to study female aggression. We would like to highlight that our experimental setup was designed to maximize the amount of female aggression observed within our current constraints. The setup was not designed to optimize tracking ability, which led to a variety of quality-control issues, such as the presence of shadows, lack of contrast, and occasional movement of the camera. We imagine that other labs establishing new setups with the explicit purpose of using automated tracking software could get much better results.

In the timing experiment, we used both manual and automated scoring to quantify female aggression in the form of headbutts. The first issue we noticed was that there was always some level of aggression recorded (i.e. in no videos were 0 frames of aggression recorded), although there were multiple instances where no aggression occurred within a pair (see Fig. 3 for how the tracking software treated videos with no aggression). When using the most conservative estimate of headbutting from the tracking software, we found that the tracking software recorded on average 12.54 ± 2.95 seconds less time spent headbutting for mated females and 9.56 ± 4.15 seconds less for virgin females than a manual scorer. We observed the same pattern (of the tracking software recording less aggression) whether we took the most or least conservative tracking estimate of aggression duration. These results suggest that the classification software is missing instances of headbutting in our videos. This may be due to errors in training the machine learning algorithm, poor tracking quality leading to issues at the classification stage, or variation in headbutting behavior that makes it difficult for the algorithm to apply universal rules to classify female aggression. However, it is generally better to be more conservative in scoring behaviors by missing behaviors rather than scoring behaviors that do not occur.

As females fight over food, we found this was a significant problem for our use of automated tracking. Our arena consisted of a circular arena with a white background and a small patch of regular fly food in the middle. When females were on the white background (Supporting Figure 7), the tracking software had few problems accurately detecting the location and size of the fly (although shadows and lighting shifts were still an issue). When the flies were on the food cap, where the majority of female aggressive encounters take place, the tracking software struggled to detect the flies accurately. We suspect this is due to a lack of contrast between the flies and the background, which makes it more difficult to detect them compared with a white or backlit background. This seems like a difficult problem to overcome for the study of female aggression as females fight over food for feeding and potentially for oviposition. In previous tests, we found that females did not display aggression or normal feeding behavior if not presented with a food patch in the arena. It is possible that the use of a micro-capillary tube, instead of a food patch with a yeast center, in the side or middle of the arena may function as an alternative limited food resource, reducing some of the issues we found.
