# Supplementary material for: Temporal and genetic variation in female aggression after mating
Source: PLoS One. 2020 Apr 29;15(4):e0229633. doi: 10.1371/journal.pone.0229633 (PMC7190144; doi:10.1371/journal.pone.0229633)
Supplement: Attachment — Submitted filename: Reviewer comments.docx [file pone.0229633.s011.docx]

Manuscript ID: PONE-D-20-03858

**“Temporal and genetic variation in female aggression after mating”**

Please find our response to reviewer comments in bold below.

**Editor comments:**

Thank you for submitting your manuscript to PLOS ONE. After careful consideration, we feel that it has merit but does not fully meet PLOS ONE’s publication criteria as it currently stands. Therefore, we invite you to submit a revised version of the manuscript that addresses the points raised during the review process.

Please respond thoroughly to the comments below.  Regarding the w1118 points, to the best of my understanding  visual impairment in these flies appears to be age dependent, but please respond to these comments as well.

**We thank the editor for this comment. They are correct that the degree of visual impairment in *w^1118^* flies increases with age – i.e. older flies are more impaired (Ferreiro et al. (2018) *Frontiers in Neuroscience*). As our flies were young (5 days old when fought), this is at the stage of the minimum difference between *w^1118^* and our other fly genotypes, although *w^1118^* flies are still visually impaired at this stage in life. We have highlighted that *w^1118^* flies are visually impaired (lines 114 & 430) and rephrased a line in the Discussion which said they were “essentially blind”, which is not strictly true at 5 days old (line 430).**

**Reviewer comments:**

Reviewer #1: This manuscript by Bath et al., reports on the temporal variation of female aggression after mating in Drosophila melanogaster. Aggression, as a behavior itself and as driven by evolutionary mechanisms, is of considerably interest to many in the behavioral neuroscience community. The paper is very well written with an informative introduction and clear hypotheses and outcomes. The authors take care in describing both the strengths and weaknesses of their automated system. The conclusion that Drosophila female aggression increased in females between two and four hours after mating and remain elevated for at least a week is very interesting. With these results and recent studies including Wang et al., Nature Feb 2020, separate experiments examining neurons and circuits responsible for the increase in encounter duration upon mating should be doable and exciting. However, resolution of the following points are required.

**We thank the reviewer for their comments and their positive evaluation of our paper.**

Major points:

1. The number of matings and fights that have been generated for this manuscript is much appreciated, however, the sole reliance on contest duration weakens the ability to draw conclusions. The authors need to provide headbutt number comparisons as well. This should be in the contest duration anyway. Encounter duration is fine but there is an element of subjectivity in this calculation – when is the cut-off that females have “stopped interacting”? The females may be across from each other eating the yeast paste without a true separation but also not aggressively interacting. Providing the simple headbutt number and statistical analysis of this number needs to be included.

**We thank the reviewer for this detailed comment. To address their comment about the cut-off for the end of an encounter, we have included a more detailed description of how we measured the end of an interaction (lines 255-259), including when one fly left the food cap, when the flies were separated by one body length, and when they stopped touching for at least three seconds.**

**In relation to the point about contest duration and headbutt number, we have shown in previous work that contest duration and headbutt number are related (Bath et al. (2017) *Nature Ecology & Evolution*), but agree that it adds more to the manuscript to include both measures. We have adjusted the methods (lines 263-4, 294, 302) and the results to reflect this. We have included new analyses of headbutt number as suggested by the reviewer (for new results: see lines 331-5, 343-347, 369-373). The results of the headbutt number analyses follow the same patterns as contest duration, except in one case where a marginally non-significant main effect in contest duration was non-significant in headbutt number (lines 331-335). Including these analyses did not change any of our interpretations or discussion of results.**

2. w1118 males have not been used to the best of my knowledge since Hoyer et al., 2008. This is because as described in this paper, “the eyes are visually impaired”, [refs within, Hengstenberg and Gotz, 1967, Wehner et al., 1967]. Currently, the background of many transgenic males are “cantonized” to eliminate this situation. The authors should either remove the w1118 data or explain that these females are visually impaired.

**To our knowledge, w1118 flies are not commonly used in aggression studies any more. However, the *w^1118^* background is often used as a background in which to introgress various types of insertions, particularly using the popular GAL4/UAS system. In a quick search, we found multiple papers from recent years that mention using *w^1118^* flies in their abstract for molecular, neuronal, or behavioural research (e.g. Ferreiro et al. (2018) *Frontiers in Neuroscience*; Qiu et al. (2017) *Comparative Biochemistry and Physiology Part A*; Xiao & Meldrum (2017) *PLOS One*). We imagine there are many more studies that do not mention the genotypes of flies used in their abstract that also use *w^1118^*. We believe that *w^1118^* is still widely used enough for this data to be interesting and important to include in our paper, with the caveats we have included that the flies are visually impaired.**

**We raise at various points during our results and discussion the fact that *w^1118^* flies are visually impaired (see lines 113-4 & 430-443). We have now added this as a prediction in our Introduction as well to highlight this from the beginning.**

3. What Fig. 2b represents isn’t clear to me and the only mention in the text is line 335. Please explain – is this headbutt only data?

**Figure 2.b is the automated tracking and classification output for the contests in the timing experiment (the same videos that were manually scored in Fig 2.a). This is solely headbutt encounter duration (as it is in Fig 2.a). We included the manual and automated scoring values next to each other so the readers could directly compare the different methods of scoring of the same videos. We have edited the figure legend for clarification (lines 380-81).**

3. Maybe I missed this but what is the size of the contest arena? This is necessary for others to replicate the assay.

**We have now added this (and corrected the size of the food cap, which was incorrect). See lines 238-9.**

Minor points:

1. Reference formatting on lines 52-54

**Fixed – this was due to an error by the referencing software that we didn’t pick up (new line 52).**

2. It would help to provide examples of when the authors hypothesize aggression will be the most beneficial to females, line 74.

**We have added examples of different species that show increased female aggression in response to different phases of reproduction (lines 73-78).**

3. Drosophila has a powerful model organism for the study of male aggression for decades and more than one reference should be given, line 81.

**We have added two more studies to support this statement. Both are reviews which discuss neuronal or physiological pathways which regulate aggression in male *Drosophila* and other species (Miczek et al. (2007) *Journal of neuroscience*; de Almeida et al. (2005) *European Journal of Pharmacology*) (line 89).**

4. w1118 should be italicized (white gene) and 1118 should be superscript as it is the allele of this gene.

**This has been corrected throughout the manuscript and the Supporting Information.**

5. Line 133, lunging is a male aggressive pattern, it makes sense to use a female pattern here.

**Fixed - we have replaced ‘lunging’ with ‘headbutting’ (line 146).**

6. Line 135, automated analysis of male aggression has been described for years, do the authors mean female here?

**We did mean female aggression and think that that is written in both lines, so are not sure exactly where the reviewer is referring to. We think the author is referring to new line numbers 149-150, which state that the development of an automated tracking and classification system would be beneficial for the study of female aggression.**

7. If the authors software can detect differences in the speed of virgin vs mated headbutts that would be amazing. Headbutts are very fast and there is a second fly involved as with male lunging giving each pair pattern a level of uniqueness.

**We agree! This would be a very exciting discovery, which is why we are continuing to develop a better setup for recording and studying female aggression, and why we have included a discussion of the issues we faced in our Supplementary Material for others trying to answer similar questions.**

8. Is there a reason Fig. 4 is described in the Discussion and not in the Results section?

**As Figure 4 was more of a general schematic of the timing of different post-mating responses rather than results from our experiments, we thought it fit more naturally in the Discussion section where we discuss the timing of different responses in mated females. It could potentially be moved if the editors think that is preferable.**

Reviewer #2: Bath et al. show that female aggression over food (measured as duration of headbutt behavior) increases following mating and persists for at least a week. This increase is robust and not dependent on the genotype of the female, or the genotype of the male she mates with. In addition, the authors have developed an automated tracking system and discuss its performance and challenges.

The authors articulate clear hypotheses and address them with carefully designed and executed experiments that are rigorously evaluated. The data support the conclusions the authors draw.

The discussion of the newly developed tracking system and some of its weaknesses is careful and will be of great value for further optimization of the automated approach.

**We thank the reviewer for their comments and positive evaluation of our paper.**
